# Orthodontic radiology: development of a clinical practice guideline

**DOI:** 10.1007/s11547-020-01219-6

**Published:** 2020-05-27

**Authors:** Aldin Kapetanović, Barbara C. M. Oosterkamp, Antoon A. Lamberts, Jan G. J. H. Schols

**Affiliations:** 1 Department of Dentistry - Orthodontics and Craniofacial Biology, Radboud Institute for Health Sciences, Radboud University Medical Centre, Nijmegen, The Netherlands; 2 Knowledge Institute of the Federation of Medical Specialists, Utrecht, The Netherlands

**Keywords:** Orthodontics, Radiology, Guideline development, Decision-making

## Abstract

**Objectives:**

Radiographs are considered essential in orthodontics. However, their diagnostic value and indications for use are still uncertain, while exposure to radiation carries health risks. This study aimed to report on the development of a clinical practice guideline on orthodontic radiology.

**Methods:**

A Guideline Development Taskforce was set up. The GRADE methodology was used for development and the RIGHT Statement for reporting of the guideline. We systematically reviewed articles to address the main clinical question: how different types of radiographs contribute to orthodontic diagnosis, treatment planning and post-treatment outcome evaluation. After a literature search and data extraction, we formulated conclusions and assessed the strength of the evidence according to the GRADE method. Both literature conclusions and the most important considerations, such as patient preferences, organizational matters and expert opinions were taken into account to finally issue recommendations.

**Results:**

7 clinical questions focused on orthopantomograms, lateral cephalograms, hand-wrist radiographs, peri-apical radiographs, bitewings, antero-occlusal radiographs, and cone-beam computer tomographic imaging. The literature search lead to 484 unique studies, of which 17 were included in the analysis. The strength of evidence of the conclusions was graded low or very low. We formulated considerations and took them into account when issuing the 13 clinical recommendations to address the clinical questions.

**Conclusions:**

There was a considerable lack of scientific evidence on this topic. Nonetheless, this guideline provides clinicians with a tool for decision-making regarding radiographic records while enhancing patient radiation protection. More research of higher quality is recommended for a future update.

**Electronic supplementary material:**

The online version of this article (10.1007/s11547-020-01219-6) contains supplementary material, which is available to authorized users.

## Introduction

Radiographs are considered essential for orthodontic treatment. Among other indications, radiographs and cephalometric analysis are used for assessing the interrelationships among the maxillofacial skeleton, dentition, and soft tissues [1, 2].

However, diagnostic value of orthodontic radiographs and indications for their use are still debatable, and studies that investigated the validity of cephalometric analysis and its influence on orthodontic diagnosis and treatment planning showed inconsistent results. Moreover, the minimum set of records required for orthodontic diagnosis and treatment planning has never been solidly established or defined in the literature [1–6].

At the same time, exposure to radiation is associated with health risks for patients and staff. These risks, more specifically, the somatic stochastic effects, are widely acknowledged and no radiation exposure, even in small doses, is free of risk, particularly in children [3, 4]. Consequently, the use of radiation by orthodontists is accompanied by a responsibility to ensure appropriate indication. It must always be justified and delivered in doses ‘as low as reasonably achievable’ [4, 5].

Due to the lack of evidence, there is an on-going discussion on the diagnostic value of orthodontic radiographs and indications for their use [2, 4]. In the past, several institutions, such as the European Commission, the British Orthodontic Society, and the American Academy of Oral and Maxillofacial Radiology, have published guidelines for orthodontic radiography [1, 7, 8]. However, a well-structured methodological approach for writing guidelines had not been applied. The now available Grading Recommendations, Assessment, Development, and Evaluation (GRADE) methodology meets all the criteria set out in the AGREE II instrument [9, 10]. Furthermore, the first reporting tool for writing practice guidelines in health care, the Reporting Items for Practice Guidelines in Healthcare (RIGHT) Statement, was published recently, in 2017 [11].

Therefore, the present study aims to report on the development of a clinical practice guideline (CPG) with recommendations for orthodontic radiology. By providing more clarity on the contribution of different types of radiographs to orthodontic diagnosis and treatment planning, and consequently their indication, the aim is both to help clinicians in their decision-making process, and protect patients from unnecessary and potentially harmful exposure to radiation.

## Materials and methods

The Dutch Association of Orthodontists initiated and financed the development of this guideline. The Knowledge Institute of the Federation of Medical Specialists provided methodological support. In this report, we followed the RIGHT statement for reporting practice guidelines [11]. The guideline was developed during the period of October 2015 to March 2018.

Firstly, a Guideline Development Taskforce was set up consisting of six members: 5 renown and experienced orthodontists working in the Netherlands, and one orthodontic resident. None of the Taskforce members reported conflicts that would preclude participation in this effort. The Dutch Patient Federation was also involved to promote the patient perspective. A methodological advisor of the Knowledge Institute of Medical Specialists supported the Taskforce to ensure an evidence-based guideline development. This guideline was developed using the GRADE methodology, in accordance with the requirements stated in the report “Medical Specialist Guidelines 2.0” published by the Quality Council of the Federation of Medical Specialists [10, 12]. This paper was prepared according to the RIGHT Statement [11]. The Taskforce established the clinical questions revolving around one main question: how different radiographs contribute to orthodontic diagnosis, treatment planning and post-treatment outcome evaluation.

### Guideline development and literature search strategy

A broad literature search was developed. Two electronic databases, Medline (OVID) and Embase, were searched, based on 40 specific search terms (for the detailed search strategy see: Supplementary file S1). The search strategy was developed by a specialized librarian of the Knowledge Institute of the Federation of Medical Specialists. Two Taskforce members (A.K. and B.O.) then selected the articles, independently of each other, based on predefined criteria. The inclusion criteria were:

- full-text article available in Dutch or English
- published between 1985 and October 2015
- study population eligible for orthodontic treatment
- study design: primary comparative research (randomized controlled trials or observational studies) or systematic reviews
- description of the stated clinical question
- description of the diagnostic modality

Studies on patients with cleft lip and palate or craniofacial anomalies were excluded.

The first selection was based on the title and abstract. The remaining articles were screened based on the full text and content. Differences between the two observers were discussed and resolved by consensus. Additionally, the references of the included articles were reviewed manually. The selected articles were then used to address the clinical questions.

### Data extraction and quality assessment

Data extraction was conducted by one Taskforce member (A.K.) and confirmed by the methodologist (A.L.). The data extracted included the study reference, study characteristics, patient characteristics, intervention, comparison/control, issues regarding follow-up, outcome measure, and effect size.

To estimate the risk of bias, a member of the Taskforce (A.K.), supported by the methodologist (A.L.), systematically assessed the individual studies. These assessments were conducted with validated instruments recommended by the Cochrane Collaboration, including the Cochrane Risk of Bias Tool for randomized controlled trials (2009) [13], and ACROBAT-NRSi for observational research (now called ROBINS-I) [14], adapted in concordance to the Cochrane Handbook for systematic reviews of interventions [15].

### Summarizing the literature and assessment of the strength of scientific evidence

For each clinical question and outcome measure, we made one or more conclusions, which summarized the scientific evidence. The strength of the evidence was determined on an outcome-by-outcome basis, according to the GRADE methodology, carried out in consensus by a member of the Taskforce (A.K.) and the methodologist (A.L.) [10, 16]. Based on five criteria (risk of bias, inconsistency, indirectness, imprecision, and publication bias), GRADE distinguishes four levels of quality for scientific evidence: high, moderate, low, and very low. These gradations refer to the degree of certainty about the literature conclusion [10].

### Considerations and recommendations

The full Taskforce then prepared a list of considerations, including patient values and preferences, costs, resource availability, organizational matters, and expert opinions, and formulated recommendations in consensus. Following the GRADE methodology, the literature conclusions, their GRADE rating and these considerations should be taken into account when issuing recommendations, even more so when the scientific evidence is weak or lacking [10].

Both the GRADE rating and the weight assigned to the considerations determined the strength of the recommendation, after weighing all relevant arguments. According to the GRADE methodology, a low level of evidence for the literature conclusions does not exclude a strong recommendation, and conversely, a weak recommendation might be given, even when the strength of scientific evidence is high [9].

### Commentary and authorization phase

The drafted guideline was submitted for comments to all concerned national dental and health insurance associations, the Dutch Patients’ Federation, the Dutch Health and Youth Care Inspectorate (IGJ), the Academic Centre for Dentistry Amsterdam (ACTA), and the University Medical Centre Groningen (UMCG). Their comments were collected, discussed and answered by the Taskforce. Based on these comments, the drafted guideline was adapted and finalized by the Taskforce.

## Results

### Establishing the clinical questions

Based on the central question the Taskforce established 7 clinical questions (CQs), one per type of radiograph, on the contribution to orthodontic diagnosis, treatment planning and post-treatment outcome evaluation of CQ#1 an orthopantomogram (OPT), CQ#2 a lateral cephalogram (LC), CQ#3 a hand-wrist (HW) radiograph, CQ#4 a peri-apical (PA), CQ#5 a bitewing (BW), CQ#6 an antero-occlusal (AO) and CQ#7 a cone-beam computed tomography (CBCT).

### Literature search

**Fig. 1:**
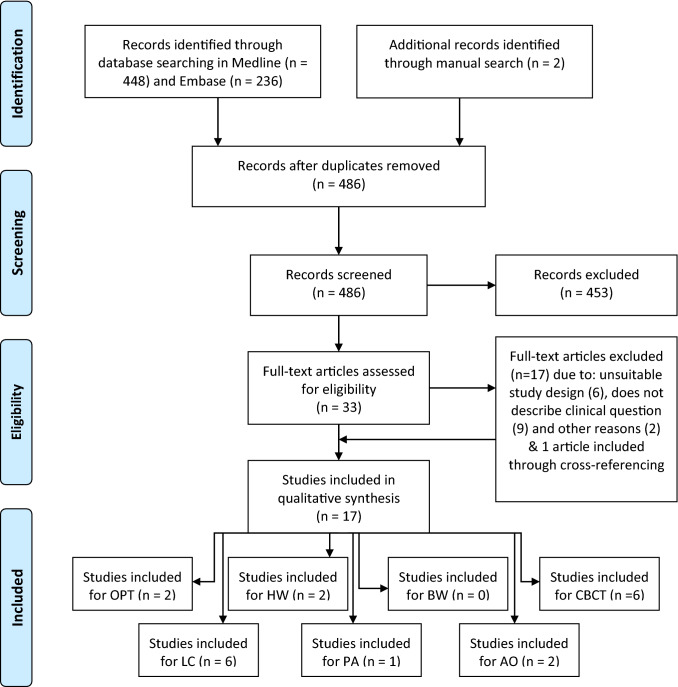
Literature search PRSIMA flowchart. *OPT* orthopantomogram, *LC* lateral cephalogram, *HW* hand-wrist radiograph, *PA* peri-apical radiograph, *BW* bitewing radiograph, *AO* antero-occlusal radiograph, *CBCT* cone-beam computed tomography. 17 studies were included in the qualitative analysis of which 2 studies addressed more than one clinical question

The literature search was performed in October 2015. It generated 484 unique hits, and two more articles were added after manual review of article references (see flow chart in Fig. 1). Based on the titles and abstracts, 453 articles were excluded. The remaining 33 full-text articles were reviewed, and 17 studies were excluded (see: Supplementary file S2). Based on cross-referencing, one additional study was included [17]. The final selection included 17 studies: [3, 5, 6, 17–30] 15 observational studies [3, 5, 18–30] and 2 RCTs [6, 17] and no systematic reviews. Two studies were included for two different clinical questions/radiographs [18, 19]. The 17 included studies were then categorized by type of radiograph for further analyses. The relevant data were placed in data extraction tables (see: Supplementary file S3) and the risk of bias was estimated (see: Supplementary file S4). Below, the literature analysis results are described per clinical question, and the assigned GRADE is given. The GRADE assessments are shown in the GRADE table (see: Supplementary file S5). According to the GRADE method RCTs start off with GRADE level HIGH and can be downgraded one or more levels. Observational studies start off with GRADE level LOW. If there is no downgrading, the level remains LOW, but if any downgrading occurs, it becomes VERY LOW. Upgrading is possible, but is rare and did not occur [10].

### Literature conclusions and assessment of the strength of scientific evidence

#### CQ#1: OPT

Two studies were included [18, 19] that considered two outcome measures. Bruks et al. [18] evaluated the additional value of LC and OPT, supplemented with a PA of the upper and lower anterior teeth for orthodontic diagnosis and treatment planning. Mattick et al. [19] examined whether significant dental abnormalities were likely to be overlooked in the anterior maxillary region, when only an OPT was used during an initial orthodontic assessment. The literature conclusions and level of evidence, according to GRADE, for the two outcome measures were:

1.1 *Adapting a diagnosis and treatment plan, based on OPT*

An OPT mainly confirmed a previous orthodontic diagnosis, based on a clinical examination, extra- and intra-oral pictures, and dental casts. However, when the diagnosis was modified based on an OPT, there was a fair chance that the treatment plan also had to be adapted. *GRADE: VERY LOW* [18].

1.2 *Detecting tooth anomalies, based on OPT*

Only a very small number of premaxilla anomalies were missed with OPT. *GRADE: LOW* [19].

#### CQ#2: LC

Six studies were included [3, 5, 6, 18, 20, 21] that considered four outcome measures. The study of Bruks et al. [18] was described above, in CQ#1. Devereux et al. [3] investigated the influence of LCs on orthodontic treatment planning. Durão et al. [5] evaluated the impact of an additional LC for orthodontic diagnosis and treatment planning. Nijkamp et al. [6] assessed the influence of an LC and cephalometric analysis on orthodontic treatment planning. Pae et al. [20] assessed the role of an LC in evaluating patients with a Class I with mild crowding, a Class II Division 2, a Class III, an open bite, or bimaxillary protrusion. Song et al. [21] assessed the reliability of ranking and grading treatment outcomes by experienced orthodontists, based on three information sources: LC, dental casts, and facial photographs, and the contribution of each to the post-treatment outcome evaluation. The literature conclusions and GRADE were:

2.1 *Change of treatment plan, based on LC*

In the general treatment planning process not involving a specific decision such as extraction/non-extraction, an LC did not appear to provide significant added value to other orthodontic records. *GRADE: VERY LOW* [6, 18].

2.2 *Change of treatment plan involving an extraction/non*-*extraction decision, based on LC*

The use of an LC in addition to other orthodontic records appeared to influence the treatment decision for extraction. This influence was most pronounced for Class II malocclusions and in patients with bimaxillary protrusion. *GRADE: VERY LOW* [3, 5, 20].

2.3 *Change of treatment plan involving growth modification, based on LC*

The use of an LC in addition to other orthodontic records did not lead to changes in the treatment plan regarding growth modification. *GRADE: VERY LOW* [3, 20].

2.4 *Post*-*treatment outcome evaluation, based on LC*

When performing a post-treatment evaluation of the results, more specifically of incisor inclination and jaw relationship, the diagnostic value provided by a combination of dental casts and facial photographs and by a combination of dental casts and LC was equivalent to the diagnostic value of a combination of dental casts, facial photographs, and LC. These results implied that the LC was not needed for these post-treatment outcome evaluations. *GRADE: VERY LOW* [21].

#### CQ#3: HW

Two studies were included [22, 23] that considered two outcome measures. Alhadlaq and Al-Shayea [22] investigated the validity of a newly proposed angular CVM method, based on measurements of the cervical vertebral bodies on an LC, versus the skeletal age determined with Greulich and Pyle’s radiographic atlas [31] or a skeletal maturation assessment, based on Fishman’s skeletal maturity indicators [32, 33] on an HW. Al Khal et al. [23] evaluated the validity of the CVM method as an indicator of skeletal age by correlating the CVM with the HW maturation method, in an attempt to eliminate the need of an HW for maturity assessments. The literature conclusions and GRADE were:

3.1 *Skeletal maturity in boys, based on HW vs LC*

There was good agreement on skeletal maturity based on information derived from an LC compared to information derived from an HW in boys. *GRADE: VERY LOW* [22, 23].

3.2 *Skeletal maturity in girls, based on HW vs LC*

There was good agreement on skeletal maturity based on information derived from an LC compared to information derived from an HW in girls. *GRADE: VERY LOW* [23].

#### CQ#4: PA

One study was selected [19] that considered one outcome measure. This study was described above, for CQ #1. The literature conclusions and GRADE were:

4.1 *Detection of anomalies, based on PA*

Anomalies in the premaxilla were missed only in a very small number of cases, when an OPT was not supplemented with PA radiographs. *GRADE: LOW* [19].

#### CQ#5: BW

No studies could be included. Accordingly, no literature conclusion could be made.

#### CQ#6: AO

Two studies were included [24, 25] that considered two outcome measures. Giles and Taylor [24] investigated whether an OPT required a supplementary AO for orthodontic diagnosis of root morphology. Witcher et al. [25] examined whether OPTs provided reliable data for judging the anterior maxilla. The literature conclusions and GRADE were:

6.1 *Root morphology, based on AO*

There seemed to be no correlation between an AO and an OPT when determining root morphology. It remains unclear which of these radiographs should be preferred for the diagnosis of root morphology. *GRADE: VERY LOW* [24, 25].

6.2 *Detection of impacted canines, based on AO*

There was a positive correlation between AO and OPT identifications of impacted canines. However, this correlation was not sufficiently strong to justify the use of either radiograph separately, without losing diagnostic information. *GRADE: VERY LOW* [25].

#### CQ#7: CBCT

Based on the literature analysis, two sub-questions for CBCT were formulated:

- *CQ#7.1. What is the diagnostic value of CBCT compared to OPT for assessing orthodontically*-*induced external apical root resorption (EARR), for localizing impacted canines, and for assessing root resorption caused by impacted canines?*
- *CQ#7.2. What is the diagnostic value of CBCT compared to PA for assessing orthodontically*-*induced EARR and iatrogenic damage to teeth during inter-radicular miniscrew placement?*

Six studies were included [17, 26–30]: 4 addressed CQ#7.1 [26, 28–30] and considered five outcome measures (7.1.1–7.1.5), and two addressed CQ#7.2 [17, 27] and considered two outcome measures (7.2.1 and 7.2.2). Alqerban et al. [26] compared the diagnostic accuracies of OPT and CBCT for the localization of impacted canines and the detection of canine-induced root resorption of maxillary incisors. Dudic et al. [28] compared OPT and CBCT in evaluating both the presence/absence and the severity of orthodontically-induced EARR near the end of orthodontic treatment with fixed appliances. Lai et al. [29] determined the diagnostic value of OPT in patients with impacted maxillary canines, assessed by a group of orthodontists and oral surgeons. They also quantified the subjective need and reasons for taking an additional CBCT. Wriedt et al. [30] evaluated whether CBCT was superior to OPT for the assessment of impacted maxillary canines. Several parameters were analyzed, including canine position and adjacent tooth resorption. De Freitas et al. [27] evaluated the frequency of orthodontically–induced EARR detected on PAs and CBCTs. Kalra et al. [17] compared the accuracies of inter-radicular miniscrew placement, with the help of either CBCT or PA and a surgical guide. The literature conclusions and GRADE were:

#### CQ#7.1

7.1.1. Detection of EARR, based on CBCT vs OPT

Less teeth with EARR were diagnosed with OPT than with CBCT. *GRADE: VERY LOW* [28].

7.1.2. Severity of EARR, based on CBCT vs OPT

It is unclear whether OPT and CBCT provided significantly different diagnoses of EARR severity. *GRADE: VERY LOW* [28].

7.1.3. Localization of impacted canines, based on CBCT vs OPT

Some evidence showed that impacted canines were more likely to be diagnosed as buccally located and less likely to be diagnosed as palatally located, based on CBCT compared to OPT. *GRADE: VERY LOW* [26, 29, 30].

7.1.4 Detection of root resorption caused by impacted canines, based on CBCT vs OPT

The number of teeth diagnosed with root resorption caused by impacted canines was underestimated with OPT, compared to CBCT. *GRADE: VERY LOW* [26, 29].

7.1.5 Severity of root resorption caused by impacted canines, based on CBCT vs OPT

The severity of lateral incisor root resorption caused by impacted canines was underestimated with OPT, compared to CBCT. It was unclear whether this difference was statistically significant. *GRADE: VERY LOW* [26].

#### CQ#7.2

7.2.1 Detection of EARR, based on CBCT vs PA

More teeth with root resorption were diagnosed with PA than with CBCT. *GRADE: LOW* [27].

7.2.2 Accuracy of inter-radicular miniscrew placement, based on CBCT vs PA

CBCT proved to be more accurate than PA for inter-radicular miniscrew placement, but the differences were small and marginally clinically relevant. *GRADE: LOW* [17].

### Commentary and authorization phase

Based on the conclusions from the literature study and the Taskforce-determined considerations, the clinical recommendations were drafted. The ensuing guideline was submitted for comments to the concerned professional associations. After reviewing the 138 comments, and adjusting the guideline where necessary, the Taskforce filed the guideline for authorization. In March 2018, the Dutch Association of Orthodontists authorized the final guideline [34].

## Discussion

Together with the literature conclusions, the considerations in the following discussion led to the final recommendations.

### CQ#1: orthopantomogram

The Taskforce considered the OPT very useful in orthodontic practice, because it provides a complete overview of the dentition, roots, and the neighboring anatomical structures [18, 19]. However, one issue was not discussed in the literature. The Taskforce estimated medico-legal reasons for taking an OPT after treatment not as a valid argument because the patient will not benefit from that X-ray. The most recent OPT taken during treatment is equally adequate for that purpose. The Taskforce therefore issued a recommendation on this topic, in the absence of evidence, which is possible and consistent with the followed methodology [9].

### CQ#2: lateral cephalogram

The use of an LC is controversial in the literature. The other diagnostic records appeared to be sufficient for treatment planning. However, in some cases, an LC and cephalometric analysis were merited [35]. The LC was the only radiograph used for post-treatment outcome evaluation, more specifically of incisor inclination and jaw relationship, even though its contribution did not appear essential [21]. In the future, 3D fusion models of 3D extra-oral stereophotogrammetric images and dental scans, might replace the LC, but these methods require further development [36, 37].

### CQ#3: hand-wrist radiograph

The HW is limited to assessing skeletal maturity only. Scientific data have indicated that skeletal maturity assessments performed on an LC are as good as assessments performed with the HW method [38].

### CQ#4: peri-apical radiograph

The Taskforce consensus was that the PA provided a more detailed view of teeth compared to the OPT. However, in most cases, the view offered by an OPT provided sufficient information [19].

### CQ#5: bitewing

No studies were identified that investigated the contribution of a BW. In the absence of evidence, we therefore issued a recommendation based on the consideration that the indication for the BW was dental rather than orthodontic diagnosis.

### CQ#6: antero-occlusal radiograph

The Taskforce consensus was that the AO offered a detailed view of the premaxilla. In most cases, the view offered by OPT provided sufficient information; nevertheless, the AO can supplement the OPT when a more detailed view is needed for the premaxilla region.

### CQ#7: cone–beam computed tomography

A 3D view from CBCT will always provide more information than a 2D view. However, this additional information should contribute substantially to a better orthodontic diagnosis and treatment plan to justify the much higher exposure to radiation [8].

When assessing the location of impacted canines, the CBCT is easier to interpret, but often unnecessary. This is also the point of view of the American Association of Orthodontists [39].

For root resorption, the cause must be differentiated. Significant EARRs can be clearly diagnosed with an OPT or PA [17, 28]. However, root resorption of the lateral incisors caused by impacted canines is more difficult to diagnose with an OPT and a CBCT with a limited field of view could be justified [40]. Furthermore, a CBCT can be justified when difficulties arise in moving impacted canines, or when the exact 3D location needs to be estimated for an operation. These conclusions were consistent with the SEDENTEXCT report by the European Commission [8]. However, the additional exposure to ionizing radiation and the higher cost of a CBCT should be discussed with the patient in advance.

## Clinical recommendations

Based on the literature conclusions and the considerations, following recommendations were issued and formulated according to the RIGHT methodology [11].

### *CQ#1*: OPT

- Consider taking an OPT for orthodontic diagnosis and treatment planning when evaluating root resorption, tooth anomalies, and treatment progress. S*trong recommendation, very low*-*quality evidence.*
- Do not take an OPT just before or after removing an orthodontic appliance at the end of active treatment. *Strong recommendation, absence of evidence.*

### *CQ#2:* LC

- Take an LC when information about skeletal dimensions and relationships is needed, and other diagnostic records do not provide sufficient data. *Strong recommendation, very low*-*quality evidence.*
- Take an LC when information about the position or inclination of the upper or lower incisors is needed, and other diagnostic records do not provide sufficient data. *Strong recommendation, very low*-*quality evidence.*
- Consider taking an LC before, during, and after treatment, when it is necessary to assess the orthodontic and/or orthopedic effect of the treatment. *Weak recommendation, very low*-*quality evidence.*

### *CQ#3:* HW

- Do not take an HW when skeletal maturity needs to be determined. An LC should be taken instead. *Strong recommendation, very low*-*quality evidence.*

### *CQ#4:* PA

- Take a PA only when the available radiographs do not provide sufficient information about a localized problem that has been diagnosed. *Strong recommendation, very low*-*quality evidence.*

### *CQ#5:* BW

- Do not take a BW for orthodontic diagnosis and treatment planning. Refer the patient to the dentist, when dental caries is suspected. *Strong recommendation, absence of evidence.*

### *CQ#6:* AO

- Consider taking an additional AO only when other available radiographs do not provide sufficient detailed information about the premaxilla. *Medium strong recommendation, very low*-*quality evidence.*

### *CQ#7:* CBCT

- Consider taking a CBCT to detect and evaluate the severity of root resorptions caused by impacted canines, when other available radiographs do not provide sufficient information, and when in doubt about the prognosis of potentially resorbed teeth. *Medium strong recommendation, very low*-*quality evidence.*
- Consider taking a CBCT to diagnose the localization of impacted canines only when other available radiographs do not provide sufficient information. *Medium strong recommendation, very low*-*quality evidence.*
- Do not take a CBCT to detect or evaluate the severity of EARR because the CBCT has no added value in comparison with an OPT. *Strong recommendation, very low*-*quality evidence.*
- Do not take a CBCT for inter-radicular miniscrew placements, because the CBCT has no added value in comparison with a PA radiograph. *Strong recommendation, low*-*quality evidence.*

## Limitations of the guideline and recommendations for future research

Several challenges were encountered during the guideline development process. The existing uncertainties regarding the use of radiographs for orthodontic purposes have led to the assessment of a wide scope of radiographs and a wide amount of data and information. It proved impossible to reduce variety without losing important information. Nevertheless, we found a limited number of studies dealing with the contribution of radiographs to orthodontic diagnosis, treatment planning and post-treatment outcome evaluation. We opted to only include articles in English and Dutch, thus, setting a limitation. We acknowledge that the inclusion of more literature would be beneficial for the guideline, yet, at the time of writing, this was the only available literature that we could retrieve, fitting the predefined criteria for its development.

Furthermore, comparisons between the studies that qualified for inclusion were complicated by methodological differences. The outcome measures were derived from the selected literature. Hence, not all possible indications for the selected radiographs were mentioned. This is especially true for CBCT, even though we were able to include significantly more studies than for the other radiographs, with the exception of the lateral cephalogram. To assess the strength of evidence, we used the GRADE method [10]. The vast majority of evidence was graded ‘very low’. Given the lack of high-quality studies, the considerations determined by the Taskforce gained weight when coming to the final recommendations, and while the strength of the evidence from the literature was weak some strong recommendations were issued. This is consistent with the methodology that was followed, even though it may seem counterintuitive [9, 10].

Clinical circumstances and considerations, however, may vary in different settings and clinicians are encouraged to reflect critically. When there are well-founded arguments, it is possible to diverge from the guideline, as the aim is to offer clinicians a guide, hereby not intending to impose limitations.

We therefore hope that this guideline will encourage more high-quality research in this field and that by the time of the update of the guideline, scheduled 5 years after publication, more literature will be available.

Moreover, as the focus of this guideline was on the contribution of radiographs, and thus their justification, we did not aim to conduct an extensive study on radiation levels. We acknowledge, though, that limiting radiation is very useful when possible, for instance by limiting the field of view [40]. When two radiographs yield comparable diagnostic information or contribute equally to the treatment, the technique with the lowest radiation is clearly preferable [8]. Nowadays, there are also techniques to limit the area of exposure for an LC by covering the cranial base and thyroid gland with protective elements [41]. These are radiologic aspects that could be additionally elaborated on in a future update of this guideline.

Guideline development has been a time-intensive process, involving many actors and stakeholders, with a duration of approximately 3 years, including the final search in October 2015 and publication. We have therefore redone the literature search on 25th of March 2020 for an estimation of newly available literature (see: Supplementary file S6). This search has revealed an interesting evolution. There was a particular increase in studies assessing CBCT, which is not entirely unexpected, being one of the more recent but also more controversial radiographic modalities. It has shown varying insights and conclusions. Pico et al. [42] stated in 2017 that further research is necessary to prove when CBCT has a clear advantage over conventional 2D radiographs. In 2019, Björksved et al. [43] concluded that “panoramic radiographs could be considered good enough for rendering palatally displaced canine position when the need for 3D information is not crucial for treatment planning”. In their systematic review, De Grauwe et al. [44] stated that “CBCT is justified only in those cases where conventional radiography fails to provide a correct diagnosis of pathology and that therefore, it cannot be regarded as a standard method of diagnosis”, however conceding that “CBCT may be justified when it positively affects treatment options or provides treatment optimization”. On the other hand, Portelli et al. [45] judged that “CBTC is fundamental for the diagnosis and treatment planning of impacted canines”, but added that more research is needed to prove the greater reliability compared to 2D radiographs. Subsequently, there still seems so be some uncertainty about the justification of CBCT and we can reaffirm our recommendation for more high-quality research being recommendable.

Moreover, except for “National guidelines for dental diagnostic imaging in the developmental age” by Firetto et al. [46], which includes a limited chapter on orthodontics, this search has not yielded any other guideline specifically on orthodontic radiology. The current guideline is, thus, the most recent one to date in the orthodontic field.

## Conclusion

The lack of evidence-based literature on orthodontic radiology led to a CPG where considerations played a significant role. Nevertheless, it is a step forward in providing clinicians with a tool for decision-making regarding acquisition and usage of radiographs, reducing variation and enhancing radiation protection of patients while not depriving the orthodontist of necessary diagnostic information. More research in this field is strongly recommended for a future update.

## Electronic supplementary material

Below is the link to the electronic supplementary material.

Supplementary material 1 (DOCX 19 kb)

Supplementary material 2 (DOCX 18 kb)

Supplementary material 3 (DOCX 68 kb)

Supplementary material 4 (DOCX 49 kb)

Supplementary material 5 (DOCX 26 kb)

Supplementary material 6 (DOCX 24 kb)
