# Supplementary material for: Orthodontic radiology: development of a clinical practice guideline
Source: Radiol Med. 2020 May 27;126(1):72–82. doi: 10.1007/s11547-020-01219-6 (PMC7870627; doi:10.1007/s11547-020-01219-6)
Supplement: Supplementary file 1 — Supplementary material 1 (DOCX 19 kb) [file 11547_2020_1219_MOESM1_ESM.docx]

**Supplementary file S1**

Search strategy for Medline (OVID) and Embase

| **Data base** | **Search terms** |
| --- | --- |
| Medline (OVID) | 1 exp Radiography, Panoramic/ or (panoramic* adj2 radiograph*).ab,ti. or ((exp Cephalometry/ or cephalomet*.ab,ti.) and lateral.ab,ti.) or Radiography, Dental/ or periapical radiograph*.ab,ti. or exp Cone-Beam Computed Tomography/ or cbct.ab,ti. or (cone beam computer and tomograph*).ab,ti. (25259)  2 exp Orthodontics/ or orthodontic*.ab,ti.  3 1 and 2 (3829)  4 limit 3 to (yr="1985 -Current" and (dutch or english)) (3193)  5 exp "Sensitivity and Specificity"/ or (Sensitiv* or Specific*).ti,ab. or (predict* or ROC-curve or receiver-operator*).ti,ab. or (likelihood or LR*).ti,ab. or exp Diagnostic Errors/ or (inter-observer or intra-observer or interobserver or intraobserver or validity or kappa or reliability).ti,ab. or reproducibility.ti,ab. or (test adj2 (re-test or retest)).ti,ab. or "Reproducibility of Results"/ or accuracy.ti,ab. or Diagnosis, Differential/ or Validation Studies.pt. (4954372)  6 treatment planning.ab,ti. (17093)  7 5 or 6 (4964083)  8 4 and 7 (922)  9 (meta-analysis/ or meta-analysis as topic/ or (meta adj analy$).tw. or ((systematic* or literature) adj2 review$1).tw. or (systematic adj overview$1).tw. or exp "Review Literature as Topic"/ or cochrane.ab. or cochrane.jw. or embase.ab. or medline.ab. or (psychlit or psyclit).ab. or (cinahl or cinhal).ab. or cancerlit.ab. or ((selection criteria or data extraction).ab. and "review"/)) not (Comment/ or Editorial/ or Letter/ or (animals/ not humans/)) (251818)  10 (exp clinical trial/ or randomized controlled trial/ or exp clinical trials as topic/ or randomized controlled trials as topic/ or Random Allocation/ or Double-Blind Method/ or Single-Blind Method/ or (clinical trial, phase i or clinical trial, phase ii or clinical trial, phase iii or clinical trial, phase iv or controlled clinical trial or randomized controlled trial or multicenter study or clinical trial).pt. or random*.ti,ab. or (clinic* adj trial*).tw. or ((singl* or doubl* or treb* or tripl*) adj (blind$3 or mask$3)).tw. or Placebos/ or placebo*.tw.) not (animals/ not humans/) (1521828)  11 Epidemiologic studies/ or case control studies/ or exp cohort studies/ or Controlled Before-After Studies/ or Case control.tw. or (cohort adj (study or studies)).tw. or Cohort analy$.tw. or (Follow up adj (study or studies)).tw. or (observational adj (study or studies)).tw. or Longitudinal.tw. or Retrospective*.tw. or prospective*.tw. or consecutive*.tw. or Cross sectional.tw. or Cross-sectional studies/ or historically controlled study/ or interrupted time series analysis (2474025) |
| Embase | 'panoramic radiography'/exp OR 'panoramic radiography' OR (panoramic*:ab,ti AND radiograph*:ab,ti) OR (lateral:ab,ti AND cephalomet*:ab,ti) OR (hand*:ab,ti OR wrist*:ab,ti OR anteroposterior:ab,ti AND radiograph*:ab,ti) OR 'periapical radiograph':ab,ti OR ('cone beam computer':ab,ti AND technolog*:ab,ti) OR cbct:ab,ti OR (3d OR 'three dimensional' AND radiograph*:ab,ti) OR 'cone beam computed tomography'/exp OR 'cone beam computed tomography'  AND ('orthodontics'/exp OR orthodontic*:ab,ti) AND [1985-2015]/py AND ([dutch]/lim OR [english]/lim)  AND (('sensitivity and specificity'/de OR sensitiv*:ab,ti OR specific*:ab,ti OR predict*:ab,ti OR 'roc curve':ab,ti OR 'receiver operator':ab,ti OR 'receiver operators':ab,ti OR likelihood:ab,ti OR 'diagnostic error'/exp OR 'diagnostic accuracy'/exp OR 'diagnostic test accuracy study'/exp OR 'inter observer':ab,ti OR 'intra observer':ab,ti OR interobserver:ab,ti OR intraobserver:ab,ti OR validity:ab,ti OR kappa:ab,ti OR reliability:ab,ti OR reproducibility:ab,ti OR (test NEAR/2 're-test'):ab,ti OR (test NEAR/2 'retest'):ab,ti OR 'reproducibility'/exp OR accuracy:ab,ti OR 'differential diagnosis'/exp OR 'validation study'/de OR 'measurement precision'/exp OR 'diagnostic value'/exp OR 'reliability'/exp) OR ‘treament planning’:ab,ti)  AND (('meta analysis'/de OR cochrane:ab OR embase:ab OR psychlit:ab OR cinahl:ab OR medline:ab OR (systematic NEAR/1 (review OR overview)):ab,ti OR (meta NEAR/1 analy*):ab,ti OR metaanalys*:ab,ti OR 'data extraction':ab OR cochrane:jt OR 'systematic review'/de) NOT ('animal experiment'/exp OR 'animal model'/exp OR 'nonhuman'/exp NOT 'human'/exp)  OR ('clinical trial'/exp OR 'randomization'/exp OR 'single blind procedure'/exp OR 'double blind procedure'/exp OR 'crossover procedure'/exp OR 'placebo'/exp OR 'prospective study'/exp OR rct:ab,ti OR random*:ab,ti OR 'single blind':ab,ti OR 'randomised controlled trial':ab,ti OR 'randomized controlled trial'/exp OR placebo*:ab,ti) NOT 'conference abstract':it)  OR 'clinical study'/de OR 'case control study'/de OR 'family study'/de OR 'longitudinal study'/de OR 'retrospective study'/de OR ('prospective study'/de NOT 'randomized controlled trial'/de) OR 'cohort analysis'/de OR (cohort NEAR/1 (study OR studies)):ab,ti OR (case:ab,ti AND (control NEAR/1 (study OR studies)):ab,ti) OR (follow:ab,ti AND (up NEAR/1 (study OR studies)):ab,ti) OR (observational NEAR/1 (study OR studies)):ab,ti OR (epidemiologic NEAR/1 (study OR studies)):ab,ti OR ('cross sectional' NEAR/1 (study OR studies)):ab,ti) |
