# Supplementary material for: Orthodontic radiology: development of a clinical practice guideline
Source: Radiol Med. 2020 May 27;126(1):72–82. doi: 10.1007/s11547-020-01219-6 (PMC7870627; doi:10.1007/s11547-020-01219-6)
Supplement: Supplementary file 2 — Supplementary material 2 (DOCX 18 kb) [file 11547_2020_1219_MOESM2_ESM.docx]

**Supplementary file S2**

**Exclusion table: 33 full-text studies were assessed for eligibility after the first screening. Out of those, 17 articles were excluded with reasons. The column clinical question shows for which clinical question, and thus radiograph, the study was selected originally.**

| **First author and year** | **Clinical question** | **Reason for exclusion** |
| --- | --- | --- |
| Bjerklin, 2006 | 7 | Unsuitable study design |
| Botticelli, 2011 | 7 | Unsuitable study design |
| Dudic, 2008 | 4 | Does not describe the clinical question |
| Durao, 2013 | 2 | Unsuitable study design |
| Ferguson, 1992 | 1 | Does not describe the clinical question |
| Haney, 2010 | 6 & 7 | Unsuitable study design |
| Katsnelson, 2010 | 1 | Does not describe the clinical question |
| Le, 2011 | 1 | Insufficient description of outcome measure |
| Lund, 2010 | 7 | Unsuitable study design |
| Patel, 2008 | 7 | Does not describe the clinical question |
| Pittayapat, 2014a | 7 | Does not describe the clinical question |
| Pittayapat, 2014b | 7 | Unsuitable study design |
| Rischen, 2013 | 7 | Does not describe the diagnostic modality |
| Signorelli, 2016 | 7 | Does not describe the clinical question |
| Tymofiyeva, 2010 | 1 | Does not describe the clinical question |
| Van Vlijmen, 2012 | 7 | Does not describe the clinical question |
| Zhou, 2008 | 2 | Does not describe the clinical question |
