# Supplementary material for: Orthodontic radiology: development of a clinical practice guideline
Source: Radiol Med. 2020 May 27;126(1):72–82. doi: 10.1007/s11547-020-01219-6 (PMC7870627; doi:10.1007/s11547-020-01219-6)
Supplement: Supplementary file 3 — Supplementary material 3 (DOCX 68 kb) [file 11547_2020_1219_MOESM3_ESM.docx]

**Supplementary file S3: data extraction table.**

**Study and patient characteristics, intervention and comparison/control, follow-up and outcome measures and effect size were extracted for each included study. The clinical question, and thus radiograph, for which the study qualified is also indicated.**

| **Study reference & Clinical question** (first author, year, CQ#) | **Study characteristics** | **Patient characteristics** | **Intervention** | **Comparison / control** | **Follow-up** | **Outcome measures and effect size** |
| --- | --- | --- | --- | --- | --- | --- |
| Al Khal, 2008  CQ #3 | Retrospective cross-sectional  Prince Philip Dental Hospital, University of Hong Kong, China  Source of funding: n.m. | Inclusion: LC & right hand HW within 1 mo, taken within circumpubertal period, HW bones appearing clearly, no systemic disease, LC clear inferior border first 4 vertebrae  Exclusion: n.m.  N=400, 200m, 200f, age range girls: 10–15y, boys 12–17y  Groups comparable at baseline? Yes | CVM evaluated on LC by Bacetti method using cervical maturation stages: CVS 1-6 | Hand-wrist maturation evaluated on HW by Fishman’s method using skeletal maturation indicators: SMI 1-11 | n.a. | Skeletal maturity in boys  Spearman's rank-correlation between CVS & SMI: r=0.9206 (significant)  Skeletal maturity in girls  Spearman's rank-correlation between CVS & SMI: r=0.9363 (significant)  For both boys & girls: linear relationship  - intra-observer variability: 25 random RX re-evaluated after 3 weeks  - inter-observer reliability: same 25 RX evaluated by another orthodontist (no results reported) |
| Alhadlaq, 2013  CQ #3 | Retrospective cross-sectional  Orthodontic  Clinic, College of Dentistry, King Saud University, Riyadh, Saudi Arabia  Source of funding:  Partly funded by the College of Dentistry Research Center, King Saud  University, Riyad | Inclusion: standardized high quality LC & left hand HW, documented patient date of birth, age max 3 months apart from absolute value of each chronological age group  Exclusion: illness affecting normal growth, orthodontic treatment, trauma to face or HW region, congenital or acquired malformation of cervical vertebrae or HW area, developmental abnormalities due  to syndromes or hormonal disorders  N=197, only males, age range: 10-15y  Groups comparable at baseline? Yes | CVMS 1-5 used to define Cervical Vertebral Maturation Angular (CVMA) Stages. Skeletal age assessment by CVMA stages for all patients | Skeletal age assessment by Greulich & Pyle’s atlas, Fisherman’s SMI stages, Baccetti’s CVS stages 1-5 | n.a. | Skeletal maturity in boys  Spearman's rank-correlation between CVMA & skeletal age: r=0.94, p<0.001  Correlation between CVMA & SMI: r=0.94, p<0.001 |
| Alqerban, 2011  CQ #7 | Retrospective observational  Division of Orthodontics, Katholieke Universiteit Leuven, Belgium  Source of funding: n.m. | Inclusion: consecutive patients with impacted or ectopically erupting maxillary canines, OPT & CBCT present, obtained within max. 2 weeks  Exclusion: n.m.  N=89 impacted maxillary canines from 60 patients, 23m, 37f, age range: 6.3-28.9y, mean: 13.2y, SD: 4.2  Groups comparable at baseline? Yes | 3 experienced dentists & 8 postgraduates analyzed 2 sets (A and B) of OPT & CBCT:  A = OPT & Accuitomo CBCT (small FOV),  Variables; canine crown width and follicle width, root development, canine angulation and location, contact between canines & incisors, root resorption severity & location | Same 3 dentists & 8 postgraduates analyzed 2 groups of sets of OPT & CBCT:  B = OPT & Scanora CBCT (medium FOV): same variables. | n.a. | Detection of root resorption caused by impacted canines  - Overall agreement I_2_: Set A no resorption: CBCT: 46.1%, OPT: 70.6%, p=0.0201. Set B no resorption: CBCT: 49.1%, OPT: 69.3%, p<0,001  - Overall agreement I_1_: Set A no resorption: CBCT: 84.9%, OPT: 87.0%, p=0.045. Set B no resorption: CBCT: 95.1%, OPT: 94.5%,  - Kappa-values for I_2_: CBCT A: 0.24, CBCT B: 0.26, OPT 0.26 & for I_1_: CBCT A: 0.63, CBCT B: 0.36, OPT: 0.23  Severity of root resorption caused by impacted canines  Slight, moderate or severe. For both lateral and central incisors p=0.020  Localisation of impacted canines  Buccally, palatally, in line of arch. Statistically sign. difference for set A: p=0.0074 & set B: p=0.0008 |
| Bruks, 1999  CQ #1+2 | Observational  Department  of Orthodontics Örebro, Sweden  Source of funding: n.m. | Inclusion: Angle Class I & II malocclusion, no previous orthodontic treatment  Exclusion: n.m.  N=70 consecutive adolescents: 33m, 37f, mean age: 13.3y (SD 1.85y, range 9.8-18.8y)  Groups comparable at baseline? n.a. | Clinical examination, diagnosis & treatment planning by orthodontic residents (4)  Records used: 1) dental casts, extra- and intraoral photographs, clinical information and 2) If necessary: LC & cephalometric analysis, OPT or PA of upper and lower front teeth | Diagnosis and treatment plan by 3 senior orthodontists = “gold standard” | n.a. | Sequence of ordering RX, change of diagnosis and treatment plan  OPT: 67 examinations, 13 changed diagnoses, 3 changed treatment plans |
| de freitas, 2013  CQ #7 | Observational  Private orthodontic clinic, Brazil  Source of funding:  grants from  National Council for Scientific & Technological  Development | Inclusion: complete ortho records, high quality RX, pictures, dental casts, edgewise ortho TX by same orthodontist completed min 52 months earlier, no retreatment  Exclusion: n.m.  N=1392 teeth from 58 patients, 28m, 30f, mean age 12y4m (SD = 2.31),  Groups comparable at baseline? Yes | 3 orthodontists analyzed PA at T1 (before fixed ortho TX), T2 (after fixed ortho TX) & T3 (52 to 288m after TX) to detect external apical root resorption (EARR) by Levander and Malmgren modified scoring system | Same 3 orthodontists analyzed CBCT at T3 for EARR using the same system | Loss-to-follow-up: 87 CBCT (ca. 6%), 124 PA at T1 and 131 PA at T2 (ca. 9%)  Reasons: extracted or congenitally absent teeth, teeth with periapical lesions, trauma & image overlapping | Detection of orthodontically induced external apical root resorption:  Frequency (%) of teeth group most affected with EARR: Maxilla: Anterior: CBCT: 22.7%, PA: 19.2% p>0.05; Premolars: CBCT: 10.8%, PA: 5.3% p<0.05; Molars: CBCT: 6.7%, PA: 4.3% p>0.05  Mandible: Anterior: CBCT: 21.1%, PA: 17.6 % p>0.05; Premolars: CBCT: 7.1%, PA: 3.1% p<0.05; Molars: CBCT: 7.3%, PA: 3.8% p<0.05 |
| Devereux2011  CQ #2 | Prospective observational  John Radcliffe Hospital, Oxford, UK  Source of funding: Oxford Radcliffe Hospitals NHS Trust | Inclusion: Patients chosen according to type of malocclusion from cohort of orthodontic patients  Exclusion: congenital anomalies  N=6, age 12y-28y  Groups comparable at baseline? n.a. | 199 orthodontists allocated to group A, B or C filled out questionnaire with 6 questions regarding treatment planning at T1.  Group A T1+T2: all records except LC  Group B T1: all records except LC, T2: all records  Group C T1+T2: all records  All records: clinical findings, photographs of dental casts, standard clinical photographs, OPT, LC & tracing LC  Response rate T1: 75% (149 of out 199 orthos) | Same orthodontists in same groups filled out the same questionnaire at T2 (8-14 weeks after T1)  Response rate T2: 77% (114 out of 149 orthodontists)  Total respons rate: 57% (114 out of 199 ortho) | n.a. | Treatment change % involving an extraction/non-extraction decision  Overall: 19,7% changes, Group B: 24,4%, p<0,05  Treatment change % involving growth modification  Overall: 10,0% changes, Group B: 10,5%, p<0,05 |
| Dudic, 2009  CQ #7 | Prospective observational  Private orthodontic  Practice, Winterthur & department of orthodontics, University of Geneva, Switzerland  Source of funding:  Swiss National Science Foundation | Inclusion: near end of ortho TX with fixed appliances  Exclusion: n.m.  N=275 teeth from 22 patients, 14m, 8f, mean age 16.7y, range: 12.6-37.2y,  Groups comparable at baseline? Yes | Two examiners assessed separately & blindly 1 OPT and 1 or 2 CBCTs per patient: presence/ absence & degree of EARR by Levander and Malmgren scoring system | Two CBCT sizes imaging areas with super-high resolution taken with 3D Accuitomo  Null hypothesis: no difference in evaluating EARR on OPT & CBCT.  Observer agreement K= 0.63 for CBCT & K=0.46 for OPT | Loss-to-follow-up:  17 teeth for OPT (6 incisors, 4 canines, 5 premolars, 2 molars), finally 258 teeth assessed | Detection of orthodontically induced external apical root resorption:  EARR Evaluation:  All teeth: CBCT no EARR: 31%, EARR: 69%; OPT no EARR: 56.5%, EARR: 43.5%  Maxillary incisors: CBCT no EARR: 13.9%, EARR: 86.1%; OPT no EARR: 20%, EARR: 80%  No p-values  Severity of orthodontically induced external apical root resorption Descriptive data on EARR (all teeth & maxillary incisors) by Levander and Malmgren scores 0-3, differences significant for both maxilla p<0.001 and mandible p<0.002 |
| Durao, 2015  CQ #2 | Observational  Faculty of Dental  Medicine, University of Porto, Portugal  Source of funding: n.m. | Inclusion: Random selection of patients with pretreatment diagnostic records  Exclusion: n.m.  N=43, 19m, 24f, age: 10-42 y  Groups comparable at baseline? n.a. | 10 orthodontists with 5-24y experience filled out questionnaire of 12 Q:  regarding diagnosis, TX planning, years of experience at T1  T1: all records except LC  Records: photos of dental casts, standard clinical photos, digital LC & OPT | Same orthodontists filled out the questionnaire at T2 (8 weeks after T1)  T2: using all records | n.a. | Change of TX plan (extraction/non-extraction)  % of Agreement between T1&T2  For Q6 “Would you extract teeth in this patient? If yes,  which?”: 56% |
| Giles, 1997  CQ #6 | Retrospective single centre  Dental hospital radiology department, UK  Source of funding: n.m. | Inclusion: OPT & AO pairs for orthodontic assessment between April and July 1995  Exclusion: n.m.  N=100 pairs  Groups comparable at baseline? Yes | Examination of 100 OPT by 2 operators assessing absent, supernumerary or displaced teeth, incisor invagination, root resorption, pathology  Lack of clarity in the premaxilla recorded | Examination of 100 AO by 2 operators assessing same variables as OPT  AO is assumed as gold standard | n.a. | Root morphology  Findings on OPT and on AO for root resorption: 1 true positive, 2 false positive, 97 true negative, 0 false negative  Specificity: 0.98  Sensitivity: 1 |
| Kalra, 2014  CQ #7 | RCT  Department of Orthodontics, Maulana Azad Institute of Dental Sciences, Delhi, India  Source of funding: n.m. | Inclusion: Ortho TX with upper and/or lower first premolars extraction & high anchorage need  Exclusion: Mixed dentition, missing teeth, severe periodontitis,  systemic diseases  N=40 mini-implant placement sites, 24 maxilla, 16 mandibula in 13 patients, 3m, 10f, age range*:* 14 - 28 y, 8 Class I, 4 Class II/1 & 1 Class III surgical case  Groups comparable at baseline? n.m. | 20 pairs interradicular sites (left & right in same arch) randomly allocated by split mouth into CBCT group and PA group taken for ideal mini-implant placement site determination and guiding: 4 bone measurements, level of mucogingival junction & distance from arch wire used to identify center of mesiodistal space at  determined ideal height  PA group: MI placement with PA & custom-made guide providing a grid | CBCT group: pre & post placement CBCTs taken. Ideal sites as determined on  CBCT correlated clinically for correct mesiodistal MI positioning at desired height | n.a. | Accuracy of interradicular placement of miniscrews  p-values for deviations of mini-implants from ideal position between CBCT and PA groups:  - height of MI from ideal height  p=0.02  - point of MI entry, p=0.143  - tip of MI, p=0.204  - MI angulation from the ideal pat, p=0.624 |
| Lai, 2014 CQ #7 | Observational retrospective  Department of Oral Surgery and Stomatology, University of Bern, Switzerland  Source of funding:  Swiss Association of Dentomaxillofacial Radiology | Inclusion: patients with OPT & CBCT between January 01/2009 & 12/2010  Exclusion: cleft palate patients  N=60, 18m, 42f, mean age 17.3 y (8.7–70.2 y)  with 72 impacted canines of which 12 bilateral impactions  Groups comparable at baseline? n.a. | 5 orthodontists & 5 oral surgeons filled out a questionnaire based on OPTs: labiopalatal location (labial, median or palatal), root resorption on central/lateral incisors, first, and/or second premolars, degree of root resorption by Ericson and Kurol, follicle size, necessity of further CBCT investigations | 1 experienced ortho  not involved in OPT analysis evaluated CBCTs: labiopalatal location of impacted maxillary canine, incidence and degree of root resorption on adjacent teeth, size of the dental follicle of the impacted maxillary canine  CBCT findings compared with questionnaire data based on OPTs | n.a. | Detection of root resorption caused by impacted canines  Root resorption (%) on OPT & CBCT for:  - lateral incisors: CBCT: no EARR 65.23%, EARR: 34.72%; OPT: no EARR 87.50%, EARR: 12.5%  - central incisors: CBCT: no EARR 93.06%, EARR: 6.94%; OPT: no EARR 96.94%, EARR 3.06%  - first premolars: CBCT: no EARR 90.28%, EARR 9.72%; OPT: no EARR 93.88%, EARR 6.12%  - second premolars: CBCT: no EARR 98.61%, EARR: 1.34%; OPT: no EARR 99.11%, EARR 0.89%  Localisation of impacted canines  Labiopalatal location of impacted maxillary canine (%) on OPT & CBCT:  - labial: CBCT 40.32%, OPT 26.73%  - median: CBCT 15.29%, OPT 12.87%  - palatal: CBCT 44.38%, OPT 57.33%  No p-values given |
| Mattick, 1999  CQ #1+4 | Retrospective observational  Department of Child Dental Health, Newcastle Dental Hospital, UK  Source of funding: n.m. | Inclusion: all consecutive patients visiting for the first time during 2 years with available OPT & AO and/or PA  Exclusion: n.m.  N=1169, 670f, 499m, age: 8-15y (mean 11,68y, SD 2,34y)  Groups comparable at baseline? n.a. | 2 orthodontists analysed the RX at T1 and categorized dental abnormalities: Extra teeth, missing teeth, ectopic teeth, short/resorbed roots, fractured roots, other abnormal root morphology, periapical pathology, other abnormality, crown abnormality  Examiners Level of agreement very good: Kappa=0.81 | Same observers and same analysis at T2: OPT + AO/PA and categorization of dental abnormalities | n.a. | Detection of premaxillary abnormalities and agreement/discrepancy between T1/T2  Complete agreement: 90% (1057 cases), disparity 9.6% (112): 90 false positives and 22 false negatives = 1.9% not visible on OPT but visible on PA/AO |
| Nijkamp, 2008  CQ #2 | Randomized crossover study  Department of Orthodontics, Academic Centre for Dentistry  Amsterdam, The Netherlands  Source of funding: n.m. | Inclusion: Caucasian Angle Class II/1 with bilaterally >½ PW disto occlusion , age 11-14y, overjet ≥6 mm, primary lower M2 present or permanent teeth erupted, treated between 10/1994 & 3/2003 and pre-treatment records (dental casts, LC & OPT) present  Exclusion: agenesis, craniofacial or dental malformations  N=48 (power analysis), 24m, 24f  Groups comparable at baseline? n.a. | 10 orthodontic postgraduates and 4 orthodontists made orthodontic treatment plans containing a dichotomous decision on treatment planning at T1 and T2.  Diagnostic records divided in two stratified groups & assigned to combination A: dental casts only, and B: dental casts, OPT, LC & LC analysis, so T1 & T2 were combinations: AA, AB or BB | Randomized crossover procedure repeated at T3 (1 month after T2) and T4 (2 months after T2) to assess consistency of orthodontic treatment plan | n.a. | Overall change of treatment plan  Overall proportions of agreement (OPA)  for orthodontists:  AB: median = 0.50, AA: median = 0.75, BB: median = 0.50  p-value AB vs AA = 0.07  p-value AB vs BB = 0.23 |
| Pae, 2001 CQ #2 | Retrospective observational  Department of Orthodontics, School of Dental Medicine,  University of Connecticut, Farmington, USA  Source of funding: n.m. | Inclusion: Patient records from archives representing a typical patient population and including most subcategories of malocclusions  Exclusion: Patients age < 10y  N=80, 49m, 31f, mean age 16.4y ± 6.16, 81% white, 10% Hispanic, 7.5% African American, 1.3% Asian  Patients classified into 5 groups: Class III, Class II/2, Class I mild anterior crowding, open bite, bimaxillary protrusion  Groups comparable at baseline? n.a. | 16 orthodontists evaluated records for severity (diagnosis) & difficulty (TX plan) on 5-point VAS at T1: using only dental casts  Treatment options: growth modification with nonsurgical palatal expansion, 4-premolar extraction, surgery, nonextraction, surgery with extraction, any extraction other than a 4-premolar | Same patients evaluated by same examiners at T2 (min. 1 week later): dental casts & LC.  Inter- and intraexaminer reliability defined | n.a. | Change of TX plan (extraction/non-extraction)  Bimaxillary protrusion TX plan at T1: 67 4-PM extractions & 126 nonextraction  T2:127 extractions & 80 nonextraction  Class II/2 at T1: 34 4-PM extractions & 45 nonextraction  T2: 17 extraction & 51 nonextraction  = Only statistically significant difference (p-values not given)  Change of TX plan (growth modification)  Total # TX plan growth modification T1: 162 & T2: 173  = not statistically significant for any subgroup |
| Song, 2014  CQ #2 | Observational  Department of Orthodontics, School and Hospital of Stomatology, Peking University, Beijing, China  Source of funding:  Ministry  of Health, China | Inclusion: unclear.  Equal # finished cases from each collaborating center (6) and equal # Class I, Class II and Class III malocclusions.  Exclusion: n.m.  N=108 in 9 groups of 12. Each group: 4 Class I, 4 Class II, 4 Class III. 30m, 78f, 72 patients < 18y, 36 patients >18y  Groups comparable at baseline? Partially yes | 69 experienced orthodontists evaluated post-treatment records separately and in different combinations: dental casts (DC), LC & facial photographs (FP) and made 2 assessments with respect  to treatment outcome: ranking: judges ranked 12 records in each group from 1 (most favorable) to 12 (least favorable); and grading: judges divided group of 12 cases into 3 categories: satisfactory, acceptable and unsatisfactory. |  | n.a. | Evaluation of TX result  Pearson correlation (r) between DC+LC & DC+FP: 0.95, between DC+LC & DC+LC+FP: 0.96 and between DC+FP & DC+LC+FP: 0.97  R^2^ values for DC+LC & DC+LC+FP: 0.92 and for DC+FP & DC+LC+FP: 0.94 |
| Witcher, 2010  CQ #6 | Cross-sectional observational  Orthodontic department, Kingston Hospital, RI, USA  Source of funding: n.m. | Inclusion: preoperative OPT & AO taken on same day from patients between 2001 & 2007 randomized with random number generator  Exclusion: patients with previously selected records & CLP patients  N=250 OPT & AO (power analysis: 210)  Groups comparable at baseline? Yes | 250 OPTs stratified into 5 blocks of 50 OPTs assessed by 10 orthodontic postgraduates for incisor root morphology & periapical pathology, impacted teeth, supernumerary teeth or odontomes, subjective opinion whether RX  provided enough information | Same observers and same analysis for 250 AOs stratified into 5 blocks of 50 AOs | Loss-to-follow-up:  1 pair partially incomplete | Root morphology  Only a stat. sign. difference between OPT and AO for: pipette-shaped (p=0.0001) & normal roots (p=0.001)  Paired samples correlation as phi coefficients. Only a stat. sign. difference between OPT and AO for: eroded/resorbed (p=0.001) and normal roots (p=0.0001)  Detection of impacted canines  Impacted canines: AO: 149/499 (30%) & OPT: 185/499 (37%), p=0.0001  Paired samples correlation expressed as phi coefficients = 0.638, p=0.0001 |
| Wriedt, 2012  CQ #7 | Diagnostic cross-over study  Dental Clinic, Faculty of Medicine, Johannes Gutenberg University Mainz, Germany  Source of funding: n.m. | Inclusion: n.m.  Exclusion: syndromes or tooth aplasia patients  N=21 patients with 29 retained upper canines: 13 left, 16 right  Groups comparable at baseline? Yes | 26 dentists evaluated 1^st^ OPT & dental casts, 2^nd^ CBCT & dental casts, random se­quence, min. 2-week interval, for impacted canine position, root dilacerations, TX for alignment & root resorptions of adjacent teeth | 2 dentists evaluated CBCT for: impacted canine labiopalatal position, root dilacerations & contact with and resorptions in adjacent teeth = master finding as basis for comparison with other dentists’ findings | n.a. | Localisation of impacted canines  Canine root identification (%) on OPT and CBCT  - no statement: CBCT 0%, OPT 6.9%  - buccal: CBCT 28.9%, OPT 27.3%  - palatal: CBCT 40.9%, OPT 42.0%  - apical: CBCT 30.2%, OPT 23.7% |

List of abbreviations:

AO: Antero-Occlusal radiograph

CBCT: Cone-Beam Computed Tomography

CLP: Cleft-Lip Palate

CVM: Cervical Vertebral Maturation

CVMA: Cervical Vertebral Maturation-Angular

CVMS: Cervical Vertebral Maturity Stage

CVS: Cervical Vertebra Stage

DC: Dental Cast

EARR: External Apical Root Resorption

FOV: Field of View

FP: Facial Photographs

HW: Hand-Wrist radiograph

I1: central incisor

I2 : lateral incisor

LC: Lateral Cephalogram

M2: second molar

MI: Mini-Implant

n.a.: not applicable

n.m.: not mentioned

NHS: National Health Service

OPT: Orthopantomogram

PA: Peri-Apical radiograph

PM: premolar

PW: Premolar Width

RX: radiograph

SD: Standard Deviation

SMI: Skeletal Maturity Indicator

TX: treatment
