# Supplementary material for: Orthodontic radiology: development of a clinical practice guideline
Source: Radiol Med. 2020 May 27;126(1):72–82. doi: 10.1007/s11547-020-01219-6 (PMC7870627; doi:10.1007/s11547-020-01219-6)
Supplement: Supplementary file 4 — Supplementary material 4 (DOCX 49 kb) [file 11547_2020_1219_MOESM4_ESM.docx]

**Supplementary file S4**

**Risk of bias table for observational studies: non-randomized clinical trials, cohort and case-control studies**

The risk of bias was assessed through validated instruments recommended by the Cochrane Collaboration. ACROBAT-NRSi was used for observational research, now called ROBINS-I^14^.

| **First author, year of publication, Clinical Question (CQ) #** | **Bias due to a non-representative or ill-defined sample of patients?**^1^  (unlikely/likely/unclear) | **Bias due to insufficiently long, or incomplete follow-up, or differences in follow-up between treatment groups?^2^**  (unlikely/likely/unclear) | **Bias due to ill-defined or inadequately measured outcome ?^3^**  (unlikely/likely/unclear) | **Bias due to inadequate adjustment for all important prognostic factors?^4^**  (unlikely/likely/unclear) |
| --- | --- | --- | --- | --- |
| Al Khal, 2008  CQ #3 | Unclear: sample defined as number, but description of randomization missing and unclear on what sample selection criteria applied. No exclusion criteria defined. Descriptive data, a.o. mean age missing. | Not applicable/unlikely: no need for follow-up | Likely: outcome well defined but no blinding. Knowledge of SMI stage could have influenced LC tracing. This information is missing. | Unlikely/Unclear |
| Alhadlaq, 2013  CQ #3 | Unlikely | Unlikely: no need for follow-up | Likely: outcome well defined but no blinding. Knowledge of SMI stage could have influenced LC tracing. This information is missing. | Unlikely: range of statistical methods concerning important prognostic factors used. |
| Alqerban, 2011 CQ#7 | Unlikely, even though inclusion criteria and patient selection unclear | Unlikely/Unclear | Unlikely/Unclear: unclear if outcome measures are appropriate | Unlikely: prognostic factors had no important role |
| Bruks, 1999  CQ #1+2 | Unlikely. Exclusion criteria not mentioned but unlikely to have caused bias | Unlikely | Unclear**:** frequency of changed diagnosis is examiner-dependent | Unlikely |
| de freitas, 2013 CQ#7 | Unlikely, even though inclusion criteria and patient selection unclear | Unlikely: 1200+ teeth analysed is large enough sample | Unlikely | Unlikely: prognostic factors had no important role |
| Devereux, 2011  CQ #2 | Unlikely | Unlikely | Unlikely | Unlikely |
| Dudic, 2009  CQ#7 | Unlikely. Exclusion criteria not mentioned but unlikely to have caused bias | Unlikely: large sample even after 17 teeth fall-out | Unlikely/Unclear: 2 clear measurable outcomes, but unconvincing statistical analysis | Unlikely: prognostic factors had no important role |
| Durao, 2015  CQ #2 | Unlikely. Exclusion criteria not mentioned but unlikely to have caused bias | Unlikely | Unlikely | Unlikely |
| Giles, 1997  CQ#6 | Unlikely. Exclusion criteria not mentioned but unlikely to have caused bias | Unlikely: not applicable | Unclear/likely: No information on inter- and intra-operator reliability, no mention of randomization, unsure whether specificity and sensitivity are appropriate outcome measures. | Likely/Unclear: unclear whether statistical methods correct. |
| Lai, 2014  CQ#7 | Likely: analysed population selection may have introduced considerable bias | Unlikely | Unlikely | Unlikely |
| Mattick, 1999  CQ #1+4 | Unlikely. Exclusion criteria not mentioned but unlikely to have caused bias | Unlikely | Unclear: presence of abnormalities cannot be checked with gold standard | Unlikely |
| Pae, 2001  CQ #2 | Unlikely | Unlikely | Unlikely | Unlikely |
| Song, 2014  CQ #2 | Unlikely. Exclusion criteria not mentioned but unlikely to have caused bias | Unlikely | Unlikely | Unlikely |
| Witcher, 2010  CQ#6 | Unlikely | Unlikely | Unlikely | Unclear: unclear which results statistically significant. |
| Wriedt, 2012  CQ#7 | Unclear: rather small, possibly not representative population | Unlikely | Unlikely | Unlikely: only simple statistics, but adjustments not necessary |

1. Failure to develop and apply appropriate eligibility criteria: a) case-control study: under- or over-matching in case-control studies; b) cohort study: selection of exposed and unexposed from different populations.
2. 2 Bias is likely if: the percentage of patients lost to follow-up is large; or differs between treatment groups; or the reasons for loss to follow-up differ between treatment groups; or length of follow-up differs between treatment groups or is too short. The risk of bias is unclear if: the number of patients lost to follow-up; or the reasons why, are not reported.
3. Flawed measurement, or differences in measurement of outcome in treatment and control group; bias may also result from a lack of blinding of those assessing outcomes (detection or information bias). If a study has hard (objective) outcome measures, like death, blinding of outcome assessment is not necessary. If a study has “soft” (subjective) outcome measures, like the assessment of an X-ray, blinding of outcome assessment is necessary.
4. Failure to adequately measure all known prognostic factors and/or failure to adequately adjust for these factors in multivariate statistical analysis

List of abbreviations:

LC: Lateral Cephalogram

SMI: Skeletal Maturity Indicator

**Risk of bias table for randomized controlled trials**

The risk of bias was assessed through validated instruments recommended by the Cochrane Collaboration. The Cochrane Risk of Bias Tool for randomized controlled trials was used for RCTs^13^:

| **Study reference & Clinical question**  (first author, year of publication, CQ#) | **Describe method of randomisation^1^** | **Bias due to inadequate concealment of allocation?^2^**  (unlikely/likely/unclear) | **Bias due to inadequate blinding of participants to treatment allocation?^3^**  (unlikely/likely/unclear) | **Bias due to inadequate blinding of care providers to treatment allocation?^3^**  (unlikely/likely/unclear) | **Bias due to inadequate blinding of outcome assessors to treatment allocation?^3^**  (unlikely/likely/unclear) | **Bias due to selective outcome reporting on basis of the results?^4^**  (unlikely/likely/unclear) | **Bias due to loss to follow-up?^5^**  (unlikely/likely/unclear) | **Bias due to violation of**  **intention to treat analysis?^6^**  (unlikely/likely/unclear) |
| --- | --- | --- | --- | --- | --- | --- | --- | --- |
| Kalra, 2014  CQ#7 | Unlikely, randomized block design | Unlikely | Unlikely | Unclear, not possible | Unclear | Unlikely | Unlikely | Unlikely |
| Nijkamp, 2008  CQ#2 | Stratification | Unlikely: cases numbered, only gender and age given | Unlikely | Unlikely | Unlikely | Unlikely | Unlikely | Unlikely |

1. Randomization: generation of allocation sequences have to be unpredictable, for example computer generated random-numbers or drawing lots or envelopes. Examples of inadequate procedures are generation of allocation sequences by alternation, according to case record number, date of birth or date of admission.
2. Allocation concealment: refers to the protection (blinding) of the randomization process. Concealment of allocation sequences is adequate if patients and enrolling investigators cannot foresee assignment, for example central randomization (performed at a site remote from trial location) or sequentially numbered, sealed, opaque envelopes. Inadequate procedures are all procedures based on inadequate randomization procedures or open allocation schedules.
3. Blinding: neither the patient nor the care provider (attending physician) knows which patient is getting the special treatment. Blinding is sometimes impossible, for example when comparing surgical with non-surgical treatments. The outcome assessor records the study results. Blinding of those assessing outcomes prevents that the knowledge of patient assignment influences the process of outcome assessment (detection or information bias). If a study has hard (objective) outcome measures, like death, blinding of outcome assessment is not necessary. If a study has “soft” (subjective) outcome measures, like the assessment of an X-ray, blinding of outcome assessment is necessary.
4. Results of all predefined outcome measures should be reported; if the protocol is available, then outcomes in the protocol and published report can be compared; if not, then outcomes listed in the methods section of an article can be compared with those whose results are reported.
5. If the percentage of patients lost to follow-up is large, or differs between treatment groups, or the reasons for loss to follow-up differ between treatment groups, bias is likely. If the number of patients lost to follow-up, or the reasons why, are not reported, the risk of bias is unclear
6. Participants included in the analysis are exactly those who were randomized into the trial. If the numbers randomized into each intervention group are not clearly reported, the risk of bias is unclear; an ITT analysis implies that (a) participants are kept in the intervention groups to which they were randomized, regardless of the intervention they actually received, (b) outcome data are measured on all participants, and (c) all randomized participants are included in the analysis.
