# Supplementary material for: Orthodontic radiology: development of a clinical practice guideline
Source: Radiol Med. 2020 May 27;126(1):72–82. doi: 10.1007/s11547-020-01219-6 (PMC7870627; doi:10.1007/s11547-020-01219-6)
Supplement: Supplementary file 5 — Supplementary material 5 (DOCX 26 kb) [file 11547_2020_1219_MOESM5_ESM.docx]

**Supplementary file S5: GRADE table.**

GRADE table for the outcome measures with an assessment of the five factors  (risk of bias, inconsistency, indirectness, imprecision, and publication bias) determining the quality of evidence. These five factors can be possible reasons to rate down the quality of the evidence and determine the final GRADE level of evidence. The clinical question, and thus radiograph, for which the study qualified is also indicated. According to the GRADE method, as applied, randomized trials start off with GRADE level HIGH and can get rated down one or more levels. Observational studies start off with GRADE level LOW. If there is no rating down, it stays LOW, but if any rating down occurs, it becomes VERY LOW. Upgrading is possible, but rare and did not occur in this guideline^10^.

| **Clinical question (#) & outcome measure** | **Limitations in study design** | **Inconsistency** | **Indirectness** | **Imprecision** | **Publication Bias** | **GRADE level of evidence** | **References** (first author, year) | |
| --- | --- | --- | --- | --- | --- | --- | --- | --- |
| CQ#1.1 Adapting a diagnosis and treatment plan, based on OPT findings | 0 | 0 | 0 | -2  Insufficient information on outcome measure and low number of participants | 0 | VERY LOW | Bruks, 1999^18^ | |
| CQ#1.2 Detecting tooth anomalies, based on OPT | 0 | 0 | 0 | 0 | 0 | LOW | Mattick, 1999^19^ | |
| CQ#2.1 Change of treatment plan, based on LC | 0 | 0 | -1  Set of diagnostic records is different | -2  Insufficient information on outcome measure and low number of participants | 0 | VERY LOW | Bruks, 1999^18^  Nijkamp, 2008^6^ | |
| CQ#2.2 Change of treatment plan involving an extraction/non-extraction decision, based on LC | 0 | -1  There is inconsistency of results | -1  Set of diagnostic records is different | -2  Very low number of participants | 0 | VERY LOW | Devereux, 2011^3^  Durao, 2015^5^  Pae, 2001^20^ | |
| CQ#2.3 Change of treatment plan involving growth modification, based on LC | 0 | 0 | -1  Set of diagnostic records is different | -2  Very low number of participants | 0 | VERY LOW | Devereux, 2011^3^  Pae, 2001^20^ | |
| CQ#2.4 Evaluation of treatment outcome, based on LC | 0 | 0 | 0 | -1  Low number of participants | 0 | VERY LOW | Song, 2014^21^ | |
| CQ#3.1 Skeletal maturity in boys, based on HW vs LC | -1: outcome was well defined. However, there was no blinding, knowledge of the SMI stage could have potentially influenced tracing of the LC. This information is missing. | 0 | -1: CVM stages by Baccetti et al. Are not exactly the same as CVMA stages as presented by Alhadlaq, 2013 even though they are derived from the CVM stages. | 0 | 0 | VERY LOW | Alhadlaq, 2013^22^  Al Khal, 2008^23^ | |
| CQ#3.2 Skeletal maturity in girls, based on HW vs LC | -1: outcome was well defined. However, There was no blinding, knowledge of the SMI stage could have potentially influenced tracing of the LC. This information is missing. | 0 | 0 | 0 | 0 | VERY LOW | Al Khal, 2008^23^ | |
| CQ#4 Detection of anomalies, based on PA | 0 | 0 | 0 | 0 | 0 | LOW | Mattick, 1999^19^ | |
| CQ#6.1 Root morphology, based on AO | 0 | -1: large discrepancy between findings | 0 | -1: small number of events | 0 | VERY LOW | Witcher, 2010^25^  Giles, 1997^24^ | |
| CQ#6.2 Detection of impacted canines, based on AO | 0 | 0 | 0 | -1: small number of events | 0 | VERY LOW | Witcher 2010^25^ | |
| CQ#7.1.1 Detection of orthodontically induced EARR, based on CBCT vs OPT | 0 | 0 | 0 | -1: limited information about effect size and small number of patients | 0 | VERY LOW | | Dudic, 2009^28^ |
| CQ#7.1.2 Severity of orthodontically induced EARR, based on CBCT vs OPT | 0 | 0 | 0 | -1: limited information about effect size and small number of patients | 0 | VERY LOW | | Dudic, 2009^28^ |
| CQ#7.1.3 Localization of impacted canines, based on CBCT vs OPT | -1 | 0 | 0 | -1: limited information about effect size and small number of patients | 0 | VERY LOW | | Alqerban, 2011^26^  Lai, 2014^29^  Wriedt, 2012^30^ |
| CQ#7.1.4 Detection of root resorption caused by impacted canines, based on CBCT vs OPT | -1 | 0 | 0 | -1: limited information about effect size and small number of patients | 0 | VERY LOW | | Alqerban, 2011^26^  Lai, 2014^29^ |
| CQ#7.1.5 Severity of root resorption caused by impacted canines, based on CBCT or OPT | 0 | 0 | 0 | -1: limited information about effect size and small number of patients | 0 | VERY LOW | | Alqerban, 2011^26^ |
| CQ#7.2.1 Detection of orthodontically induced EARR, based on CBCT vs PA | 0 | 0 | 0 | 0 | 0 | LOW | de Freitas, 2013^27^ | |
| CQ#7.2.2 Accuracy of interradicular miniscrew placement, based on CBCT vs PA | 0 | 0 | 0 | -2: very small number of patients and not reaching clinical relevance | 0 | LOW | Kalra, 2014^17^ | |

**List of abbreviations:**

CBCT: Cone-Beam Computed Tomography

CVM: Cervical Vertebral Maturation

CVMA: Cervical Vertebral Maturation-Angular

LC: Lateral Cephalogram

OPT: Orthopantomogram

PA: Peri-Apical radiograph

SMI: Fishman’s Skeletal Maturity Indicator
