# Supplementary material for: Orthodontic radiology: development of a clinical practice guideline
Source: Radiol Med. 2020 May 27;126(1):72–82. doi: 10.1007/s11547-020-01219-6 (PMC7870627; doi:10.1007/s11547-020-01219-6)
Supplement: Supplementary file 6 — Supplementary material 6 (DOCX 24 kb) [file 11547_2020_1219_MOESM6_ESM.docx]

**Supplementary table S6**

Search strategy for Medline (OVID) and Embase from October 2015 to 25^th^ March 2020

| **Data base** | **Search terms** |
| --- | --- |
| Medline (OVID) | 1 exp Radiography, Panoramic/ or (panoramic* adj2 radiograph*).ab,ti. or ((exp Cephalometry/ or cephalomet*.ab,ti.) and lateral.ab,ti.) or Radiography, Dental/ or periapical radiograph*.ab,ti. or exp Cone-Beam Computed Tomography/ or cbct.ab,ti. or (cone beam computer and tomograph*).ab,ti. (35909)  2 exp Orthodontics/ or orthodontic*.ab,ti. (63575)  3 1 and 2 (5374)  4 limit 3 to dt=20150101-20200325 (1703)  5 limit 4 to (dutch or english) (1580)  6 exp "Sensitivity and Specificity"/ or (Sensitiv* or Specific*).ti,ab. or (predict* or ROC-curve or receiver-operator*).ti,ab. or (likelihood or LR*).ti,ab. or exp Diagnostic Errors/ or (inter-observer or intra-observer or interobserver or intraobserver or validity or kappa or reliability).ti,ab. or reproducibility.ti,ab. or (test adj2 (re-test or retest)).ti,ab. or "Reproducibility of Results"/ or accuracy.ti,ab. or Diagnosis, Differential/ or Validation Studies.pt. (6428718)  7 5 and 6 (455)  8 (meta-analysis/ or meta-analysis as topic/ or (meta adj analy$).tw. or ((systematic* or literature) adj2 review$1).tw. or (systematic adj overview$1).tw. or exp "Review Literature as Topic"/ or cochrane.ab. or cochrane.jw. or embase.ab. or medline.ab. or (psychlit or psyclit).ab. or (cinahl or cinhal).ab. or cancerlit.ab. or ((selection criteria or data extraction).ab. and "review"/)) not (Comment/ or Editorial/ or Letter/ or (animals/ not humans/)) (438723)  9 (exp clinical trial/ or randomized controlled trial/ or exp clinical trials as topic/ or randomized controlled trials as topic/ or Random Allocation/ or Double-Blind Method/ or Single-Blind Method/ or (clinical trial, phase i or clinical trial, phase ii or clinical trial, phase iii or clinical trial, phase iv or controlled clinical trial or randomized controlled trial or multicenter study or clinical trial).pt. or random*.ti,ab. or (clinic* adj trial*).tw. or ((singl* or doubl* or treb* or tripl*) adj (blind$3 or mask$3)).tw. or Placebos/ or placebo*.tw.) not (animals/ not humans/) (1964525)  10 Epidemiologic studies/ or case control studies/ or exp cohort studies/ or Controlled Before-After Studies/ or Case control.tw. or (cohort adj (study or studies)).tw. or Cohort analy$.tw. or (Follow up adj (study or studies)).tw. or (observational adj (study or studies)).tw. or Longitudinal.tw. or Retrospective*.tw. or prospective*.tw. or consecutive*.tw. or Cross sectional.tw. or Cross-sectional studies/ or historically controlled study/ or interrupted time series analysis/ [Onder exp cohort studies vallen ook longitudinale, prospectieve en retrospectieve studies] (3396845)  11 7 and 8 (15) **=** systematic reviews  12 (7 and 9) not 11 (41) = RCTs  13 (7 and 10) not (11 or 12) (168) = observational research  14 11 or 12 or 13 (224) = total |
| Embase | \| **No.** \| **Query** \| **Results** \| \| --- \| --- \| --- \| \| #11 \| #8 OR #9 OR #10 **[totaal]** \| 167 \| \| #10 \| #4 AND #7 NOT (#8 OR #9) **[observational research]** \| 110 \| \| #9 \| #4 AND #6 NOT #8 **[RCTs]** \| 41 \| \| #8 \| #4 AND #5 **[systematic reviews]** \| 16 \| \| #7 \| 'major clinical study'/de OR 'clinical study'/de OR 'case control study'/de OR 'family study'/de OR 'longitudinal study'/de OR 'retrospective study'/de OR 'prospective study'/de OR 'cohort analysis'/de OR ((cohort NEAR/1 (study OR studies)):ab,ti) OR (('case control' NEAR/1 (study OR studies)):ab,ti) OR (('follow up' NEAR/1 (study OR studies)):ab,ti) OR (observational NEAR/1 (study OR studies)) OR ((epidemiologic NEAR/1 (study OR studies)):ab,ti) OR (('cross sectional' NEAR/1 (study OR studies)):ab,ti) \| 5182534 \| \| #6 \| 'clinical trial'/exp OR 'randomization'/exp OR 'single blind procedure'/exp OR 'double blind procedure'/exp OR 'crossover procedure'/exp OR 'placebo'/exp OR 'prospective study'/exp OR rct:ab,ti OR random*:ab,ti OR 'single blind':ab,ti OR 'randomised controlled trial':ab,ti OR 'randomized controlled trial'/exp OR placebo*:ab,ti \| 3009193 \| \| #5 \| 'meta analysis'/de OR cochrane:ab OR embase:ab OR psycinfo:ab OR cinahl:ab OR medline:ab OR ((systematic NEAR/1 (review OR overview)):ab,ti) OR ((meta NEAR/1 analy*):ab,ti) OR metaanalys*:ab,ti OR 'data extraction':ab OR cochrane:jt OR 'systematic review'/de \| 488438 \| \| #4 \| #1 AND #2 AND #3 AND [1-11-2015]/sd AND ([dutch]/lim OR [english]/lim) NOT ('conference abstract'/it OR 'editorial'/it OR 'letter'/it OR 'note'/it) NOT (('animal experiment'/exp OR 'animal model'/exp OR 'nonhuman'/exp) NOT 'human'/exp) \| 349 \| \| #3 \| 'sensitivity and specificity'/de OR sensitiv*:ab,ti OR specific*:ab,ti OR predict*:ab,ti OR 'roc curve':ab,ti OR 'receiver operator':ab,ti OR 'receiver operators':ab,ti OR likelihood:ab,ti OR 'diagnostic error'/exp OR 'diagnostic accuracy'/exp OR 'diagnostic test accuracy study'/exp OR 'inter observer':ab,ti OR 'intra observer':ab,ti OR interobserver:ab,ti OR intraobserver:ab,ti OR validity:ab,ti OR kappa:ab,ti OR reliability:ab,ti OR reproducibility:ab,ti OR ((test NEAR/2 're-test'):ab,ti) OR ((test NEAR/2 'retest'):ab,ti) OR 'reproducibility'/exp OR accuracy:ab,ti OR 'differential diagnosis'/exp OR 'validation study'/de OR 'measurement precision'/exp OR 'diagnostic value'/exp OR 'reliability'/exp OR 'treament planning':ab,ti \| 7934963 \| \| #2 \| 'orthodontics'/exp OR orthodontic*:ab,ti \| 52846 \| \| #1 \| 'panoramic radiography'/exp OR 'panoramic radiography' OR (panoramic*:ab,ti AND radiograph*:ab,ti) OR (lateral:ab,ti AND cephalomet*:ab,ti) OR ((hand*:ab,ti OR wrist*:ab,ti OR anteroposterior:ab,ti) AND radiograph*:ab,ti) OR 'periapical radiograph':ab,ti OR ('cone beam computer':ab,ti AND technolog*:ab,ti) OR cbct:ab,ti OR ((3d OR 'three dimensional') AND radiograph*:ab,ti) OR 'cone beam computed tomography'/exp OR 'cone beam computed tomography' \| 62013 \| |
